# Exploratory Person-Centred Competence Profiles Among Italian Physiotherapists: Overall Competence Gradients and Occupational Correlates

**DOI:** 10.3390/healthcare14213752

**Published:** 2026-11-03

**Authors:** Emanuela Prendi, Enkeleda Gjini, Florian Spada, Blerina Duka, Blerta Qokaj, Françeska Dervishaliaj, Tommaso De Ruvo, Viktorija Xharra, Ippolito Notarnicola

**Affiliations:** 1 Faculty of Medicine, University “Our Lady of the Good Counsel”, 1000 Tirana, Albania; blertaqokaj@yahoo.com (B.Q.); f.dervishaliaj@unizkm.al (F.D.); 2 Department of Biomedical Sciences, Faculty of Medicine, University “Our Lady of the Good Counsel”, 1000 Tirana, Albania; e.gjini@unizkm.al (E.G.); f.spada@unizkm.al (F.S.); bleriduka@yahoo.it (B.D.); 3 Department of Rehabilitation, ASL Bari, 70018 Bari, Italy; tommaso.deruvo@asl.bari.it; 4 Department of Biomedicine and Prevention, University of Rome Tor Vergata, 00133 Rome, Italy; viktorijaxharra@icloud.com; 5 Department of Medicine and Surgery, University of “Kore”, Piazza dell’Università, 94100 Enna, Italy

**Keywords:** physiotherapy, professional competence, competence profiles, cluster analysis, workforce development, continuing professional development, person-centred analysis

## Abstract

**Highlights:**

**What are the main findings?**

• The retained two-cluster solution was reproducible but primarily reflected a broad overall competence gradient across the five empirical components rather than sharply contrasting domain-specific configurations.
• Self-employment and higher job satisfaction were independently associated with membership in the Higher Relative Competence profile.

**What are the implications of the main findings?**

• Person-centred clustering may complement conventional score-based summaries at the group level, but the present solution should be interpreted as exploratory segmentation of self-perceived competence rather than as evidence of naturally occurring professional categories.
• The identified profiles should be used developmentally and not for individual credentialing until they are externally replicated and validated.

**Abstract:**

Background/Objectives: Competence in physiotherapy is multidimensional, but person-centred analyses may either reveal distinct configurations or primarily reproduce differences in overall level. This secondary analysis examined whether reproducible competence groupings could be identified among Italian physiotherapists and whether they provided occupational information beyond the continuous MSPC total score. Methods: The source dataset comprised 481 physiotherapists. The recurrent eight-person response-pattern subgroup identified in higher-k solutions was excluded a priori from the primary person-centred analysis after case-level review, yielding a primary analytic sample of 473 participants; the full 481-participant analysis was retained as a sensitivity analysis. Five varimax-rotated PCA components were used for k-means clustering (k = 2–5). Cluster stability was assessed by the bootstrap-adjusted Rand index, and sensitivity analyses compared clustering with a median split of the MSPC total score and tested incremental occupational information. Results: In the primary n = 473 analysis, the retained two-group solution comprised 284 and 189 participants, showed modest separation (silhouette = 0.304; Calinski–Harabasz = 223.0; Davies–Bouldin = 1.23) and high bootstrap stability (mean ARI = 0.905; median = 0.917). The groups were broadly parallel and primarily reflected an overall competence gradient. Concordance with a strict median split at the MSPC median (108) was 91.5% (Cohen’s κ = 0.829). Self-employment remained the strongest adjusted correlate of Higher Relative Competence membership (OR = 3.33, 95% CI 2.18–5.09), while each one-category increase in job satisfaction was associated with higher odds of membership (OR = 1.37, 95% CI 1.12–1.68). For self-employment, adding cluster membership to a model already containing the continuous MSPC total score improved fit (likelihood-ratio χ^2^ = 20.25, *p* < 0.001), whereas adding the total score after cluster membership did not (χ^2^ = 1.15, *p* = 0.284). The full-sample sensitivity analysis reproduced the same substantive pattern. Conclusions: The solution is best interpreted as an exploratory, level-based segmentation of self-perceived competence rather than as qualitatively distinct professional types. Its incremental value appears strongest for employment status. Excluding the eight wording-sensitive cases from the primary analysis did not alter the central occupational finding.

## 1. Introduction

Professional competence in rehabilitation is increasingly framed as a multidimensional and context-dependent construct rather than a fixed set of technical skills. The World Health Organization Rehabilitation Competency Framework (RCF) emphasizes that high-quality rehabilitation depends on the integration of practice, professionalism, learning and development, management and leadership, and research-related capabilities [1]. In physiotherapy, recent competency initiatives similarly stress the need for a shared language of observable performance across clinical reasoning, communication, professionalism, systems-based practice, reflective learning and education [2,3,4,5].

Competence also has an explicitly developmental character. In contemporary rehabilitation frameworks, competence is not treated as a binary state that is acquired at graduation and then retained unchanged. Rather, it reflects the capacity to integrate knowledge, clinical reasoning, communication, ethical judgement, collaboration, learning and professional responsibility in response to changing patient needs and service environments. The WHO Rehabilitation Competency Framework (RCF) was developed around this premise and organizes rehabilitation practice across domains that include practice, professionalism, learning and development, management and leadership, and research [1,6]. Its mixed-methods development demonstrated broad international agreement that rehabilitation competence requires both profession-specific activities and cross-cutting behaviours that remain relevant across settings and stages of professional development [7].

This broader interpretation is particularly relevant to physiotherapy. The profession has expanded beyond a narrow treatment-oriented model and increasingly encompasses prevention, long-term condition management, advanced practice, health promotion, interprofessional coordination, digital care, education, service redesign and leadership. International work examining physiotherapy competency documents has shown substantial alignment with the WHO-RCF while also highlighting emerging expectations related to technology, entrepreneurship and social accountability [8]. Similarly, the World Physiotherapy education framework positions competence along a lifelong learning continuum, linking entry-level education with continuing professional development and continued fitness to practise [6]. These developments make the assessment of professional competence relevant not only to educational quality assurance but also to workforce planning and organizational development.

This shift is important because the contemporary physiotherapy workforce is required to respond to ageing populations, multimorbidity, complex rehabilitation pathways, technological change and increasingly autonomous forms of practice. Competence therefore develops across multiple dimensions and may not evolve uniformly within the same professional. A physiotherapist can be strong in technical-clinical practice yet less confident in reflective learning, interprofessional collaboration, patient involvement or evidence-based planning. Mean scores and regression coefficients can describe overall tendencies but may obscure these within-professional configurations.

The Multidimensional Scale of Physiotherapist Competences (MSPC) was developed to assess the breadth of professional competence in physiotherapy. Its primary validation study in 481 Italian physiotherapists demonstrated adequate internal consistency and a multidimensional structure and also identified an association between professional competence and job satisfaction [9]. That study was deliberately variable-centred: its primary aims concerned psychometric validation, correlations and prediction. It did not examine whether distinct subgroups of physiotherapists display recognizable patterns of competence across dimensions.

The original MSPC study established the psychometric properties of the instrument and examined variable-centred associations between competence and job satisfaction [9]. The present secondary analysis addresses a different question: whether the same multidimensional information organizes individuals into reproducible person-level groupings, and, critically, whether those groupings are configurationally distinct or primarily reflect an overall competence gradient. This distinction is important because a multidimensional scale can be psychometrically valid even when between-person heterogeneity is dominated by general level rather than by sharply different domain shapes. Accordingly, the value of a person-centred analysis should not be assumed a priori; it must be judged by the empirical structure that emerges and by whether that structure adds interpretable information beyond conventional score-based summaries.

This question also addresses a broader challenge in competency-based education. Competency frameworks are frequently used to define what professionals should be able to do, but translating frameworks into decisions about continuing education requires information about where competence gaps or strengths are concentrated. Recent work in rehabilitation workforce evaluation has argued that competency analysis can complement traditional workforce indicators by providing information on proficiency, task distribution and areas requiring educational investment [10]. In physical therapist residency education, competency-based assessment has similarly been used to document developmental progression across domains rather than relying only on time spent in training [11]. A profile approach extends this logic to practising professionals by examining whether multidimensional competence patterns can identify subgroups with potentially different developmental needs.

Previous person-centred competence studies in health professions have used cluster analysis or latent profile analysis to identify subgroups in digital health competence, nursing informatics, patient safety and research competence [12,13,14,15,16,17]. Some of these studies reported groups that differed in both overall level and relative domain pattern, whereas others were dominated by broad high-versus-low gradients. This distinction has direct interpretive consequences. A configuration-based solution may support domain-specific developmental hypotheses, while a predominantly level-based solution is better understood as a reproducible segmentation of overall multidimensional competence. Physiotherapy-specific evidence on this question remains limited. The present study therefore tests, rather than presumes, whether MSPC-based person-centred analysis yields qualitatively differentiated configurations.

The relevance of this approach to physiotherapy is reinforced by current developments in competency-based education and continuing professional development (CPD). Recent reviews and international frameworks have highlighted wide variation in physiotherapy competency expectations and in CPD structures, while calling for approaches that connect competency assessment to targeted professional development [2,18,19]. A profile-based analysis may therefore offer a clinically and educationally meaningful way to identify where competence is consistently high and where selected groups might benefit from tailored support.

Continuing professional development is especially important in this context because physiotherapists practise in environments in which clinical evidence, technologies, organizational expectations and professional roles evolve continuously. Recent European and international studies have documented marked variation in CPD structures, access and perceived needs across rehabilitation professions and jurisdictions [19,20]. This variation means that the same competence-development strategy may not be equally relevant to all practitioners. Profiling can provide a data-driven basis for moving from generic CPD provision toward differentiated learning pathways that build on existing strengths while addressing domain-specific needs.

Employment context may be one source of such heterogeneity. Employed and self-employed physiotherapists can differ in autonomy, responsibility for service organization, exposure to market pressures, opportunities for interprofessional collaboration, and requirements for self-directed professional development. These differences do not imply that one employment model is inherently associated with superior competence, but they provide a plausible reason to examine whether competence profiles are distributed differently across occupational arrangements. Likewise, post-basic education, age, professional experience and perceived job satisfaction may be related to profile membership through distinct mechanisms. Identifying such correlates can help generate hypotheses for longitudinal studies and targeted workforce interventions.

Conceptually, occupational context may relate to self-perceived competence through several non-exclusive pathways. Greater job autonomy may increase responsibility for clinical decisions, service organization and self-directed learning; supportive organizational environments may provide feedback, mentorship, interprofessional exposure and protected learning opportunities; and access to continuing professional development may facilitate repeated application and integration of competence across domains. Conversely, constrained roles, limited learning climate or reduced access to development opportunities may restrict opportunities to practise and consolidate broader professional capabilities. These mechanisms were not directly measured in the source dataset and are therefore treated here as theoretical explanations to be tested in future longitudinal or mixed-methods research, not as causal conclusions from the present analysis.

Accordingly, the study had three exploratory objectives: (1) to determine whether the multidimensional MSPC data yielded reproducible person-centred groupings and whether these were configurationally distinct or mainly level-based; (2) to evaluate the stability and practical adequacy of competing cluster solutions without interpreting internal stability as external validation; and (3) to examine demographic, educational and occupational correlates of membership in the higher relative competence group. Because the analysis is cross-sectional, self-reported and secondary, all occupational explanations were treated as hypotheses about association rather than causal mechanisms.

## 2. Materials and Methods

### 2.1. Study Design and Data Source

This study used a secondary, exploratory person-centred analysis of cross-sectional data collected for the MSPC validation project. The source dataset included 481 practising physiotherapists recruited in Italy through professional associations, academic mailing lists and public/private healthcare institutions. The original study design, recruitment procedures and psychometric objectives have been reported previously [9].

The reporting approach was guided by principles applicable to observational secondary analyses: transparent description of the source dataset, clear distinction between exploratory and confirmatory inference, presentation of effect sizes alongside *p*-values, and explicit discussion of limitations related to self-report and convenience sampling. Because no universally accepted reporting guideline exists specifically for k-means profile studies in health-professions competence research, the analytic decisions and model-selection criteria are described in sufficient detail to permit replication.

The present analysis was designed to extract additional information from the same dataset without duplicating the original study question. The primary unit of inference was profile membership derived from multivariate competence patterns, rather than the psychometric performance of the MSPC or the direct prediction of job satisfaction.

### 2.2. Participants

Eligible participants were active physiotherapists with ongoing professional practice. Students and trainees were excluded. The source dataset contained 481 complete responses. Case-level examination of the recurrent eight-person subgroup identified in the three- to five-cluster solutions showed a marked wording-sensitive response pattern: these participants strongly endorsed both positively and negatively worded statements before reverse scoring, making Component 2 disproportionately influential. Consistent with the reviewer’s recommendation, these eight observations were excluded from the primary person-centred analysis, leaving 473 participants. The full sample of 481 was retained for sensitivity analysis. In the primary sample, mean age was 31.2 years (SD 8.4) and mean professional experience was 7.3 years (SD 7.8); 240 participants were women, 232 were men and one identified as other. A total of 253 participants (53.5%) were employed and 220 (46.5%) were self-employed/freelance physiotherapists.

The occupational composition of the dataset offered a useful feature for the present analysis: employed physiotherapists and self-employed physiotherapists were both well represented. This permitted examination of employment status without the extreme imbalance that often limits subgroup analyses. Educational categories were retained as recorded in the source dataset to avoid post hoc reclassification that could obscure differences in post-basic academic pathways.

In the primary analytical sample (n = 473; Table 1), post-basic education comprised another degree (n = 67, 14.2%), specialist/master degree (n = 210, 44.4%), first-level master (n = 176, 37.2%), second-level master (n = 16, 3.4%) and doctorate (n = 4, 0.8%). Job satisfaction was assessed using a five-category item ranging from “not at all” to “very”.

For clustering, standardized multidimensional component scores were used to preserve relative information across competence domains and to prevent variables with larger raw variances from dominating Euclidean distance. Importantly, this choice did not assume that person-centred clustering would necessarily provide information beyond the total MSPC score. The analysis was explicitly interpreted according to the observed shape of the resulting groups: a parallel high-versus-low pattern would indicate a predominantly level-based gradient, whereas non-parallel component patterns would provide stronger evidence of distinct multidimensional configurations.

The MSPC contains 25 self-report items scored on a five-point Likert scale. Items cover technical-clinical practice, communication, collaborative and reflective behaviours, ethical-professional conduct, evidence-based decision-making, patient involvement and learning-related behaviours. Negatively worded items (items 3, 5, 7, 12, 14, 17, 22 and 24) were reverse-scored so that higher values consistently indicated higher perceived competence. Internal consistency of the 25 corrected items in the present dataset was high (Cronbach’s alpha = 0.871).

Because this secondary study focused on multivariate person-level structure, a five-component principal component analysis (PCA) with varimax rotation was used as a data-reduction step to generate orthogonal empirical competence axes for clustering. The rotated solution accounted for 55.2% of item variance. The complete rotated item-loading matrix is reported in Supplementary Table S1. The empirical axes showed partial content overlap with the five domains reported in the primary MSPC validation study [9], but they were not identical to those validated domains and should not be interpreted as replacement subscales. Notably, Component 2 was dominated by reverse-keyed items, indicating a wording-sensitive method component in addition to substantive competence content. This distinction is important because the present PCA was used only to create orthogonal inputs for clustering, whereas the previous study addressed psychometric structure and scale validation.

The five empirical components were therefore labelled descriptively according to their dominant loadings: (1) clinical-reflective and relational practice; (2) reverse-keyed self-regulation/adherence response pattern; (3) evidence-based clinical planning; (4) ethical and person-centred professionalism; and (5) patient-centred adaptability and learning. Factor signs are arbitrary; for presentation they were oriented so that the Higher Relative Competence group had positive component means. The second component’s concentration of reverse-keyed items is treated as a methodological limitation and is considered explicitly when interpreting the recurrent eight-person subgroup.

### 2.3. Person-Centred Cluster Analysis

Rotated component scores were standardized to z-scores before clustering. K-means clustering with Euclidean distance was estimated for two-, three-, four- and five-cluster solutions. Each model used 100 random initializations to reduce dependence on local starting solutions.

Model selection in the primary analysis was performed after exclusion of the eight wording-sensitive observations. The two- through five-cluster solutions were compared using the silhouette coefficient, Calinski–Harabasz index, Davies–Bouldin index, minimum cluster size, interpretability and parsimony. In the n = 473 primary sample, the two-cluster solution had the strongest Calinski–Harabasz value and avoided the fragmentation seen in higher-k solutions; the three- and four-cluster solutions showed lower silhouette values, while the five-cluster solution generated a one-person class. The two-cluster solution was therefore retained as the most parsimonious representation of the dominant structure, while acknowledging its modest silhouette value. The original n = 481 model-selection results, including the recurrent eight-person subgroup, were retained only as a sensitivity analysis.

Bootstrap stability was used to assess whether the retained segmentation reflected reproducible structure rather than a single sample-dependent partition. The adjusted Rand index (ARI) quantifies agreement between two classifications while correcting for chance; values closer to 1 indicate greater concordance. Repeated resampling therefore provided an empirical estimate of how consistently individuals could be reassigned to comparable profile structures under sampling perturbation.

Age and years of professional experience were compared across groups using Welch independent-samples t-tests. Categorical variables were compared using chi-square tests, with Cramér’s V as an effect-size estimate. Job satisfaction was retained in the original questionnaire order from 1 (“not at all”) to 5 (“very”): “not at all”, “little”, “enough”, “satisfied”, and “very”. The placement of “enough” below “satisfied” follows the wording and ordinal sequence of the source response categories, with “enough” indicating adequacy without the explicit positive satisfaction conveyed by “satisfied”.

### 2.4. Statistical Analysis of Profile Correlates

Age and years of professional experience were compared across groups using Welch independent-samples t-tests. Categorical variables were compared using chi-square tests, with Cramér’s V as an effect-size estimate. The job-satisfaction response categories were retained in the original questionnaire order: 1 = not at all satisfied, 2 = slightly satisfied, 3 = fairly satisfied (Italian: ‘Abbastanza’), 4 = satisfied, and 5 = very satisfied. This wording clarifies that ‘Abbastanza’ represents an intermediate degree of satisfaction rather than a category semantically equivalent to ‘satisfied’.

A multivariable binary logistic regression estimated adjusted associations with membership in the Higher Relative Competence group. Predictors were employment status, gender, age (per five-year increase), years of professional experience (per five-year increase), post-basic education category and ordinal job satisfaction. The single participant identifying gender as ‘other’ was excluded from the adjusted gender model because a one-person category could not support stable estimation. Multicollinearity was assessed using the Pearson correlation between age and professional experience and variance inflation factors (VIFs) in the full adjusted model. Because age and experience were highly correlated, sensitivity models were also refitted after omitting each variable in turn.

### 2.5. Primary Exclusion and Sensitivity Analyses

The recurrent eight-person subgroup identified in the three-cluster solution on the full source dataset was examined before defining the primary analysis. Because the subgroup showed a pronounced wording-sensitive pattern—high endorsement of both positively and negatively worded items before reverse scoring, with Component 2 dominated by reverse-keyed items—the eight observations were excluded from the primary clustering and all primary profile-correlate analyses. PCA, varimax rotation, standardization, k-means model selection, bootstrap stability assessment and downstream comparisons were then re-estimated from the remaining 473 participants. The original 481-participant analysis was retained as a sensitivity analysis to assess robustness.

To evaluate whether the primary two-group classification provided information beyond overall MSPC level, the corrected 25-item MSPC total score was divided at the sample median (108), using ≤108 versus >108, and concordance with k-means membership was quantified by percentage agreement and Cohen’s kappa. Incremental occupational information was evaluated using nested adjusted models. For employment status, logistic regression models predicting self-employment compared the continuous MSPC total score, cluster membership and both predictors, adjusting for gender, age, professional experience, post-basic education and job satisfaction. For job satisfaction, proportional-odds ordinal logistic models compared the total score, cluster membership and both together, adjusting for the remaining covariates. Nested models were compared using likelihood-ratio tests.

Job satisfaction was included as an occupational correlate rather than interpreted as an outcome caused by competence profile. This distinction is necessary because the cross-sectional design does not establish temporal ordering. Similarly, self-employment was treated as a marker of occupational context rather than a causal exposure. The model was intended to identify characteristics independently associated with profile membership and to generate hypotheses for future longitudinal or mixed-methods studies.

## 3. Results

### 3.1. Primary Cluster Selection and Sensitivity Analyses

After excluding the eight wording-sensitive cases, the primary analytic sample comprised 473 participants. The two-cluster solution separated the sample into 284 participants (60.0%) and 189 participants (40.0%). Its silhouette coefficient was 0.304, the Calinski–Harabasz index was 223.0 and the Davies–Bouldin index was 1.23, indicating modest rather than strong separation. In the primary n = 473 dataset, the three-cluster solution no longer isolated the recurrent eight-person subgroup and showed poorer separation (silhouette = 0.282; Calinski–Harabasz = 186.8; Davies–Bouldin = 1.40; cluster sizes 216/174/83). The four-cluster solution produced sizes of 157/127/120/69, and the five-cluster solution generated a one-person class; therefore, the two-group solution remained the most parsimonious and interpretable representation of the dominant structure. Bootstrap stability remained high (mean ARI = 0.905; median = 0.917; 2.5th–97.5th percentile interval = 0.792–0.983).

The median MSPC total score remained 108. Using a strict median split (≤108 vs. >108), 91.5% of participants received the same low/high classification as in the primary two-cluster solution, with Cohen’s κ = 0.829 (246 concordant lower, 187 concordant higher and 40 discordant). This confirms that the retained grouping is predominantly level-based rather than configurationally distinct.

The full-sample sensitivity analysis (n = 481), which retained the eight wording-sensitive observations, reproduced the previously reported 287/194 partition (silhouette = 0.314; mean bootstrap ARI = 0.89) and the same substantive employment-status association. Thus, exclusion of the eight cases changed the numerical details of the primary solution but not the central interpretation.

### 3.2. Description of the Two Relative Competence Groups

The first group was labelled Lower Relative Competence (n = 284, 60.0%). It showed negative standardized scores on all five empirical components: clinical-reflective and relational practice (z = −0.56), reverse-keyed self-regulation/adherence response pattern (z = −0.49), evidence-based clinical planning (z = −0.47), ethical and person-centred professionalism (z = −0.53), and patient-centred adaptability and learning (z = −0.14). The label is explicitly relative to this sample and does not indicate inadequate professional competence or objectively poor clinical performance.

The second group was labelled Higher Relative Competence (n = 189, 40.0%). It showed positive standardized scores across all five empirical components: clinical-reflective and relational practice (z = 0.85), reverse-keyed self-regulation/adherence response pattern (z = 0.73), evidence-based clinical planning (z = 0.70), ethical and person-centred professionalism (z = 0.79), and patient-centred adaptability and learning (z = 0.21). The broadly parallel direction across the five components indicates that the principal distinction remains one of overall multidimensional competence level rather than a qualitatively different domain configuration.

The parallel component profiles are therefore interpreted cautiously. They demonstrate coherent co-variation across domains and reproducible segmentation within this sample, but they do not establish distinct competence phenotypes. Internal bootstrap stability indicates that the partition is reproducible under resampling; it does not show that the groups are naturally occurring, externally valid or educationally distinct categories.

Bootstrap evaluation supported the reproducibility of the primary n = 473 solution: mean ARI was 0.905, median ARI was 0.917, and the 2.5th–97.5th percentile interval was 0.792–0.983 (Table 1).

**Figure 1:**
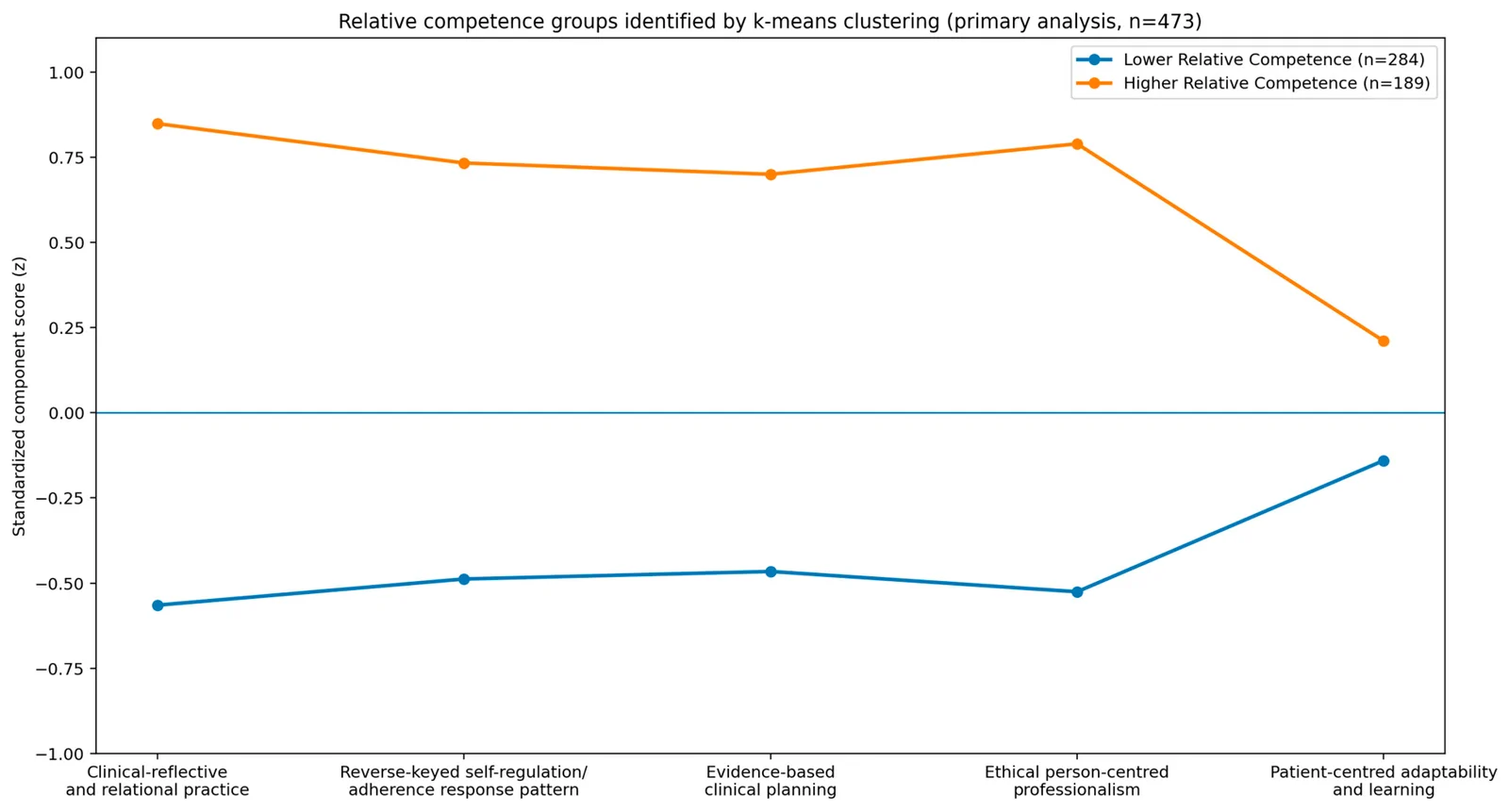
Standardized empirical-component scores for the primary two-group k-means solution after exclusion of the eight wording-sensitive cases (n = 473). Component 2 is labelled consistently as the reverse-keyed self-regulation/adherence response pattern. The broadly parallel trajectories indicate a dominant overall competence gradient rather than sharply contrasting domain-specific configurations.

The broadly parallel direction across the five components indicates that the principal distinction remains one of overall multidimensional competence level rather than a qualitatively different domain configuration (Figure 1).

### 3.3. Demographic and Occupational Characteristics by Profile

Age did not differ significantly between the Lower Relative Competence and Higher Relative Competence groups (31.1 ± 8.5 versus 31.3 ± 8.2 years; *p* = 0.807). Professional experience was also similar (7.0 ± 7.7 versus 7.8 ± 7.8 years; *p* = 0.318).

Employment status showed the largest categorical difference (chi-square *p* < 0.001; Cramér’s V = 0.33). Self-employed physiotherapists represented 66.7% of the Higher Relative Competence group compared with 33.1% of the Lower Relative Competence group. Post-basic education also differed at the unadjusted level (*p* < 0.001; V = 0.21): first-level master qualifications were more common in the Higher Relative Competence group (48.1% versus 29.9%), whereas specialist/master degree holders were more common in the Lower Relative Competence group (52.5% versus 32.3%). Gender distribution differed modestly (*p* = 0.002; V = 0.16), with men representing 58.2% of the Higher Relative Competence group and 43.0% of the Lower Relative Competence group.

The magnitude of the employment-status association was notable relative to the other observed differences. However, the cross-sectional data do not indicate whether self-employment contributes to competence development, whether physiotherapists with stronger perceived competence are more likely to enter self-employment, or whether both processes occur. The result is therefore best interpreted as evidence that occupational context and competence configuration are related and warrant more focused study.

Educational differences were more nuanced. Although the distribution of post-basic qualifications differed across profiles in unadjusted comparisons, these categories did not retain independent associations once other characteristics were considered simultaneously. This suggests that formal educational attainment alone does not fully explain the multivariate competence pattern. The content, recency and practice integration of continuing education may be more important than the existence of a qualification category, but these features were not captured in the dataset.

Job satisfaction also differed across groups (*p* = 0.001; Cramér’s V = 0.20). A “very” satisfied response was reported by 48.7% of the Higher Relative Competence group compared with 29.6% of the Lower Relative Competence group (Table 2).

**Table 2:** Participant characteristics according to competence profile.

Characteristic	Lower Relative Competence (n = 284)	Higher Relative Competence (n = 189)	*p*-Value
Age, years, mean (SD)	31.1 (8.5)	31.3 (8.2)	0.807
Professional experience, years, mean (SD)	7.0 (7.7)	7.8 (7.8)	0.318
Employment: employed, n (%)	190 (66.9)	63 (33.3)	<0.001
Employment: self-employed, n (%)	94 (33.1)	126 (66.7)	
Post-basic: other degree, n (%)	38 (13.4)	29 (15.3)	<0.001
Post-basic: specialist/master degree, n (%)	149 (52.5)	61 (32.3)	
Post-basic: first-level master, n (%)	85 (29.9)	91 (48.1)	
Post-basic: second-level master, n (%)	10 (3.5)	6 (3.2)	
Post-basic: doctorate, n (%)	2 (0.7)	2 (1.1)	
Gender: female, n (%)	162 (57.0)	78 (41.3)	0.002
Gender: male, n (%)	122 (43.0)	110 (58.2)	
Job satisfaction: not at all, n (%)	3 (1.1)	1 (0.5)	0.001
Job satisfaction: little, n (%)	14 (4.9)	5 (2.6)	
Job satisfaction: enough, n (%)	134 (47.2)	64 (33.9)	
Job satisfaction: satisfied, n (%)	49 (17.3)	27 (14.3)	
Job satisfaction: very, n (%)	84 (29.6)	92 (48.7)	

### 3.4. Multivariable Correlates of Higher Relative Competence Profile Membership

In the primary adjusted logistic regression, self-employment was the strongest independent correlate of Higher Relative Competence membership (OR = 3.33, 95% CI 2.18–5.09, *p* < 0.001). Job satisfaction was also independently associated: each one-category increase in satisfaction corresponded to a 37% increase in the odds of belonging to the Higher Relative Competence group (OR = 1.37, 95% CI 1.12–1.68, *p* = 0.002).

The adjusted analysis reinforces two points. First, neither age nor professional experience independently explained Higher Relative Competence membership, although their high correlation (r = 0.895) produced VIFs of 5.15 and 5.08, respectively; sensitivity models omitting either variable did not materially change the self-employment or job-satisfaction estimates. Second, employment status remained strongly associated after adjustment for demographic, educational and satisfaction covariates. In incremental analyses of self-employment, adding cluster membership to a model already containing the continuous MSPC total score significantly improved fit (likelihood-ratio χ^2^ = 20.25, *p* < 0.001), whereas adding the total score after cluster membership did not (χ^2^ = 1.15, *p* = 0.284). This suggests that the person-centred partition captures employment-related structure not fully represented by a simple linear total-score effect.

Gender, age, years of professional experience and the post-basic education categories were not independently associated after simultaneous adjustment. The model likelihood-ratio test was significant (*p* < 0.001), with McFadden pseudo-R^2^ = 0.120. These findings indicate association rather than causation and should not be interpreted as evidence that self-employment or job satisfaction produces higher competence (Table 3).

## 4. Discussion

This secondary analysis extends the primary MSPC validation study by asking a person-centred question, but the revised interpretation is deliberately more conservative than in the original submission. The retained two-group solution was reproducible within the sample, yet the component trajectories were broadly parallel and the silhouette coefficient indicated only modest separation. The dominant pattern is therefore best described as an overall multidimensional competence gradient rather than as two qualitatively distinct competence configurations.

The contribution of the clustering analysis should not be framed as proof that conventional total scores are insufficient. The 91.5% agreement with a median split and κ = 0.829 demonstrate that the two groups largely reproduce an overall low-versus-high relative competence gradient. The incremental analyses nevertheless add an important qualification: cluster membership contributed information about self-employment beyond the continuous MSPC total score, whereas it did not add information about job satisfaction once the total score was included. The scientific contribution is therefore narrower and more defensible than originally stated: person-centred segmentation may capture a nonlinear or pattern-based occupational signal for selected outcomes, but its added value is outcome-specific rather than universal. This outcome-specific incremental association should not be taken as evidence of distinct competence configurations: a nonlinear relationship between the continuous MSPC score and employment status, or categorization effects, may also explain the improvement in model fit. These alternatives were not directly tested.

This finding remains conceptually informative. Multidimensionality at the measurement level does not imply that practising professionals necessarily form sharply contrasting domain-specific phenotypes.

In this sample, the empirical dimensions were distinguishable as measurement axes, but between-person heterogeneity was dominated by a broad common level. This differs from person-centred studies in other competence domains that have reported more uneven or configuration-specific profiles [12,13,14,15,16,17] and suggests that physiotherapy competence development may, in this sample, be relatively coordinated across clinical-reflective, self-regulatory, evidence-based, ethical-person-centred and adaptive dimensions.

The presence of coherent cross-domain profiles is consistent with contemporary competency frameworks. The WHO RCF conceptualizes rehabilitation competence as an integration of professional behaviours, knowledge, skills and values rather than a collection of isolated technical tasks [1]. Recent physical therapy frameworks similarly include clinical practice, communication, reflective learning, professionalism, education and systems-based functions [2,3,4,5]. Our Higher Relative Competence profile is therefore conceptually consistent with the idea that advanced professional functioning requires simultaneous development across technical and non-technical domains.

The finding also resonates with recent validation work comparing physiotherapy competency documents with the WHO-RCF. Mocke and colleagues found that a large set of physiotherapy competencies could be accommodated within the RCF while identifying emerging areas that require greater attention, including technology and broader system-facing capabilities [8]. This supports the idea that competence is simultaneously multidomain and integrated. A physiotherapist who develops only procedural expertise without communication, ethical judgement, learning capacity and collaboration would not meet contemporary expectations of comprehensive professional performance.

From a workforce perspective, the analysis complements the emerging use of competency information in rehabilitation planning. Mills and colleagues showed that competency analysis can add information not captured by headcounts or labour-market measures, particularly by identifying proficiency patterns and opportunities for workforce optimization [10]. Although the present study is conducted at the individual rather than national system level, the logic is similar: a workforce cannot be understood completely from how many professionals it contains; it is also necessary to understand how competence is distributed within that workforce.

The findings also align with recent person-centred studies in other health professions. Jarva and colleagues identified high, intermediate and low digital-health competence profiles among healthcare professionals and demonstrated that profile membership was associated with professional role and practice context [20]. Kaihlanen et al. similarly found distinct nursing informatics profiles related to orientation and user proficiency [21]. Patient-safety and research-competence studies have also shown that professional groups can contain latent subpopulations with different competency patterns and different associated factors [13,14]. International cluster research published in 2026 further supports the utility of competence profiling for identifying heterogeneous workforce needs across settings and countries [12].

The strongest adjusted association in the primary analysis was employment status. Self-employed physiotherapists had more than three times the odds of belonging to the Higher Relative Competence group compared with employed physiotherapists (OR = 3.33), and the incremental model showed that cluster membership improved prediction of self-employment even after the continuous MSPC total score was included. This is the clearest evidence of added value from the person-centred analysis. Several non-exclusive mechanisms may explain the finding. Independent practice can expose physiotherapists to broader responsibility for clinical decision-making, triage, patient communication, scheduling, service organization, financial and administrative choices, professional updating and continuity of care. Such roles may create repeated opportunities to integrate competencies that are otherwise distributed across different organizational functions. Self-employment may also require more self-directed identification of learning needs and more active management of professional networks, referral relationships and patient expectations. Conversely, selection is equally plausible: physiotherapists who already perceive themselves as broadly competent, autonomous and confident may be more likely to choose independent practice. A third possibility is residual contextual confounding, because specialty area, caseload complexity, income, working hours, access to CPD, organizational climate and degree of actual autonomy were not measured. The association should therefore be interpreted as a robust occupational correlate and a hypothesis about professional context, not as evidence that self-employment causes higher competence. In particular, the improved model fit does not establish a unique multidimensional profile effect, because the association with the total MSPC score may be nonlinear.

Importantly, the finding should not be interpreted as evidence that employed physiotherapists are less professionally capable. Employment within hospitals and rehabilitation organizations may provide structured specialization, team-based expertise, formal supervision and role differentiation that are not captured by a broad self-perceived competence profile. The result instead suggests that employment arrangement may organize how competencies are experienced, integrated and self-appraised. Future longitudinal studies should measure actual decision latitude, breadth of role, CPD exposure, workload, organizational learning climate and objective performance to determine whether autonomy mediates the association, whether higher perceived competence predicts later movement into self-employment, or whether both processes operate simultaneously.

Job satisfaction remained independently associated with profile membership, even though satisfaction was not the principal outcome of this secondary study. The direction is consistent with the primary MSPC paper, in which professional competence was associated with job satisfaction [9]. However, the present person-centred analysis adds a different interpretation: satisfaction may reflect, or be reinforced by, the experience of functioning coherently across multiple professional domains. Conversely, supportive and satisfying work contexts may provide more opportunities to learn, exercise autonomy and consolidate competence. Longitudinal research is needed to examine reciprocal pathways.

The association with job satisfaction is also compatible with several non-exclusive explanations. Perceived competence may enhance professional efficacy and meaning, thereby supporting satisfaction; satisfying work environments may provide more learning opportunities and recognition, thereby strengthening perceived competence; and shared organizational factors may influence both. The primary MSPC paper found an association between competence and job satisfaction at the variable level [9], while the present person-centred analysis shows that this relationship also appears when competence is represented as a multivariate profile. The replication across analytical perspectives strengthens the descriptive consistency of the association but does not resolve causal direction.

Post-basic education showed clear differences between profiles in unadjusted analyses but did not remain independently significant in the multivariable model. This is an important finding rather than a null result to be dismissed. Competence development is influenced by both formal education and practice environments. Recent research on rehabilitation CPD has emphasized substantial variation in access, regulation and practical use of continuing professional development across Europe and globally [18,19]. Formal credentials alone may therefore be insufficient proxies for ongoing competence development. Learning opportunities, professional autonomy, feedback, workplace support and the extent to which new knowledge is used in practice may be equally important.

This result has practical relevance for continuing professional development. Formal qualifications are important, but competency development depends on whether learning is aligned with professional needs, transferred into practice and reinforced by feedback and opportunities for application. International work has highlighted unequal access to CPD and substantial variation in how rehabilitation professionals engage with ongoing development [19,20]. Consequently, future studies should collect richer information on CPD intensity, type, recency, reflective learning and workplace support rather than relying only on the highest educational credential.

The results have implications for competency-based education. Current physiotherapy scholarship increasingly argues that competence assessment should be linked to observable performance and development over time [3,4,5]. A profile-based approach can complement total scores by showing whether a professional is broadly strong across domains or whether selected components lag behind the rest. This does not mean that unsupervised cluster labels should be used for credentialing or employment decisions. Rather, competence profiles may be useful as developmental feedback at group level—for example, to design CPD pathways, mentoring or educational interventions tailored to different workforce needs.

The approach also aligns with the developmental logic of competency-based education. Recent physical therapy work has emphasized milestones and observable progression rather than time-based assumptions of competence [3,11]. Although the MSPC is a self-report instrument rather than a performance-based milestone tool, the same principle applies: assessment should inform what development is needed next. The present profiles may therefore be viewed as an exploratory bridge between psychometric measurement and individualized professional development, not as fixed categories or credentials. Because the second component was strongly influenced by reverse-keyed wording, response styles may contribute to classification even after exclusion of the eight atypical cases; accordingly, group assignment should not guide individual educational or employment decisions.

The choice of two groups in the primary n = 473 analysis deserves comment. After removing the eight wording-sensitive observations, increasing k did not reveal a more compelling substantive configuration: the three- and four-cluster solutions had lower silhouette values than the two-cluster model, and the five-cluster solution produced a one-person class. The two-group solution was therefore retained for parsimony, adequate class size and interpretability. The high bootstrap ARI supports internal reproducibility of this partition, but it should not be interpreted as evidence that the groups are naturally occurring or externally validated professional categories. The modest silhouette coefficient further indicates substantial overlap between groups.

The recurrent eight-person subgroup was treated as a data-quality and wording-sensitivity issue rather than as a substantive professional phenotype. These participants showed very high endorsement of positively worded items together with high endorsement of negatively worded items before reverse scoring, producing an extreme pattern on Component 2, which was itself dominated by reverse-keyed items. Following the reviewer’s recommendation, the eight observations were therefore excluded from the primary analysis and the complete n = 481 analysis was retained as a sensitivity analysis. Re-estimating PCA and clustering in the n = 473 sample preserved the same broad two-group competence gradient and the strong self-employment association. The full-sample sensitivity analysis reproduced the same substantive conclusions, indicating that the core findings are not dependent on these atypical response patterns.

### 4.1. Strengths and Limitations

This study has several strengths. It uses a relatively large national sample with nearly equal representation of employed and self-employed physiotherapists, a previously validated multidimensional competence instrument, multiple model-selection indices and a bootstrap assessment of cluster stability. It also explicitly distinguishes the present secondary research question from the previously published validation objectives, reducing conceptual overlap between the two papers.

Another strength is the explicit separation between psychometric validation and profile discovery. The study does not present clustering as additional evidence that the MSPC factor structure is valid; instead, it uses the multidimensional information from the instrument to answer a distinct workforce question. This reduces the risk of conflating measurement structure with person classification. The use of multiple cluster-validity criteria and bootstrap stability further reduces reliance on an arbitrary choice of k.

Limitations should be considered. First, convenience sampling limits population-level generalizability. Second, all competence data were self-reported and may be influenced by social desirability, response style, professional identity or overestimation; the groups therefore represent self-perceived competence and not verified clinical performance. Third, k-means produces sample-dependent partitions rather than naturally existing categories, and the modest silhouette coefficient indicates substantial overlap. Fourth, the five empirical competence axes were generated for data reduction and should not be interpreted as newly validated MSPC subscales. Fifth, job satisfaction was measured with a single item. Sixth, the model explained a limited proportion of profile-membership variation (McFadden pseudo-R^2^ = 0.120), indicating that important workplace and organizational variables were not captured. Finally, the cross-sectional design precludes temporal or causal inference. Furthermore, wording-related response patterns may have influenced PCA scores and cluster assignment, and no explicit nonlinear total-score model was evaluated as an alternative explanation for the incremental employment association.

Additional methodological limitations deserve emphasis. K-means assumes roughly spherical clusters in Euclidean space and provides a hard assignment of every individual to one group. It does not estimate classification uncertainty in the way latent profile analysis does, nor does it provide likelihood-based fit indices. The current solution should therefore be considered an exploratory empirical typology. Replication with Gaussian mixture models or latent profile analysis would allow direct comparison of profile probabilities, covariance structures and alternative class solutions.

The profiles are based on self-perceived competence, which is valuable for understanding professional self-appraisal but is not equivalent to observed performance. Self-ratings can be affected by response style, confidence, professional identity and social desirability. The absence of supervisor ratings, objective performance indicators or patient-level measures means that the Higher Relative Competence label should not be interpreted as verified superior clinical performance. Future research should examine convergence between profile membership and external indicators such as peer assessment, continuing education engagement, clinical outcomes or structured performance assessment.

Finally, the source dataset was not originally designed specifically for person-centred modelling. Variables that might explain profile membership—such as specialty area, workload, organizational climate, leadership, income, rurality, CPD participation, advanced practice responsibilities and perceived autonomy—were either unavailable or insufficiently detailed. Their omission likely contributes to residual unexplained heterogeneity and limits mechanistic interpretation of the employment-status association.

### 4.2. Implications for Research and Practice

Future studies should test the reproducibility of these profiles in independent Italian samples and in other European countries. Replication should preferably use confirmatory person-centred methods, including latent profile analysis, and should examine measurement invariance of the MSPC before cross-group comparisons. Longitudinal designs could determine whether professionals transition between competence profiles over time and whether CPD, mentorship, role changes or professional autonomy predict such transitions.

A logical next step is a prospective multicentre study in which profile analysis is planned a priori and the MSPC is accompanied by measures of workplace context, autonomy, learning climate and CPD behaviour. A larger and more diverse sample could support latent profile analysis with robust enumeration of classes and formal assessment of measurement invariance before profile comparison. Repeated measurement would determine whether individuals move between profiles over time and whether such transitions are associated with training, career changes or organizational interventions.

Qualitative follow-up would add complementary explanatory depth. Interviews with physiotherapists from different profiles could explore how they understand competence development, which experiences they perceive as formative, and how employment context shapes learning opportunities and professional responsibility. A mixed-methods design would be particularly useful for interpreting why self-employment is associated with the Higher Relative Competence profile and for identifying modifiable mechanisms rather than treating employment category itself as the intervention target.

For workforce development, the current findings support cautious, group-level use of competence assessment rather than automatic assignment of individuals to different interventions. A practical next step would be to use aggregate MSPC domain scores to identify areas in which lower relative competence is widespread, then pilot modular CPD packages—for example, reflective practice and communication, evidence-based clinical reasoning, or leadership and research development—and evaluate change prospectively using pre/post MSPC scores together with objective or supervisor-rated outcomes. Because the present groups mainly reflect overall level rather than contrasting domain shapes, the data do not yet justify substantively different educational pathways solely on the basis of cluster membership.

Any future application should avoid punitive appraisal or credentialing based on profile membership. Potential organizational and economic benefits—such as directing scarce CPD resources toward demonstrated group-level needs, reducing redundant generic training and improving alignment between workforce development and service priorities—remain hypotheses to be tested rather than demonstrated outcomes of this study. Likewise, possible downstream benefits for service quality or patient outcomes require prospective evaluation with external performance measures.

## 5. Conclusions

Professional competence among Italian physiotherapists showed a reproducible but predominantly level-based person-centred segmentation in the primary n = 473 analysis after exclusion of eight wording-sensitive cases. The two-group solution comprised 284 Lower Relative Competence and 189 Higher Relative Competence participants and was strongly concordant with a median split of the MSPC total score (91.5%; κ = 0.829), indicating that it should not be interpreted as evidence of qualitatively distinct professional competence types. Nevertheless, self-employment remained strongly associated with Higher Relative Competence membership, and cluster membership added information about self-employment beyond the continuous total score. The complete n = 481 sensitivity analysis yielded the same substantive interpretation. The value of person-centred profiling is therefore outcome-specific and exploratory, with its clearest incremental signal related to employment context.

The findings support person-centred analysis as an exploratory complement to conventional psychometric description, not as a replacement for continuous MSPC scoring. The association with independent work warrants targeted longitudinal investigation of autonomy, breadth of responsibility, self-directed learning and occupational selection mechanisms. Until these findings are externally replicated and linked to objective competence indicators, the groups should be interpreted as sample-dependent summaries of self-perceived competence and should not be used for individual credentialing, performance decisions or prescriptive educational assignment.

## Figures and Tables

**Table 1:** Comparison of k-means clustering solutions (k = 2–5) based on internal validity indices and cluster sizes in the primary analytic sample (n = 473).

Clusters	Silhouette	Calinski–Harabasz	Davies–Bouldin	Cluster Sizes
2	0.304	223.0	1.23	284/189
3	0.282	186.8	1.40	216/174/83
4	0.281	168.2	1.39	157/127/120/69
5	0.286	156.4	1.14	156/127/119/70/1

**Table 3:** Multivariable logistic regression for membership in the Higher Relative Competence profile.

Predictor	Adjusted OR	95% CI	*p*-Value
Self-employed vs. employed	3.33	2.18–5.09	<0.001
Male vs. female	1.23	0.81–1.87	0.332
Age, per 5-year increase	0.89	0.66–1.19	0.429
Professional experience, per 5-year increase	1.26	0.92–1.72	0.158
Specialist/master degree vs. other degree	0.66	0.36–1.23	0.190
First-level master vs. other degree	1.21	0.65–2.24	0.550
Second-level master/doctorate vs. other degree	0.58	0.19–1.74	0.333
Job satisfaction, per one-category increase	1.37	1.12–1.68	0.002

## Data Availability

The raw data supporting the conclusions of this article will be made available by the authors on request.

## Supplementary Materials

The following supporting information can be downloaded at: https://www.mdpi.com/article/10.3390/healthcare14213752/s1. Table S1: Varimax-rotated PCA item loadings used to derive the five empirical clustering components.
