# Supplementary material for: Exploratory Person-Centred Competence Profiles Among Italian Physiotherapists: Overall Competence Gradients and Occupational Correlates
Source: Healthcare (Basel). 2026 Nov 3;14(21):3752. doi: 10.3390/healthcare14213752 (PMC13762525; doi:10.3390/healthcare14213752)
Supplement: Supplementary file 1 [file healthcare-14-03752-s001.zip › healthcare-4629201-supplementary.pdf]

Table S1. Varimax-rotated PCA item loadings used to derive the five empirical clustering components.

Item	C1	C2	C3	C4	C5
**Item 1**	0.709	0.150	0.200	0.127	−0.087
**Item 2**	0.643	0.072	0.339	0.185	0.077
**Item 3**	−0.017	0.573	0.011	0.107	0.489
**Item 4**	0.049	0.119	0.696	0.106	0.067
**Item 5**	−0.147	0.655	0.109	0.063	0.224
**Item 6**	0.261	0.014	0.234	0.585	0.154
**Item 7**	0.026	0.338	−0.027	0.158	0.656
**Item 8**	0.408	−0.118	0.245	0.297	0.235
**Item 9**	0.635	0.016	0.211	0.240	0.106
**Item 10**	0.463	−0.046	0.311	0.344	0.414
**Item 11**	0.280	0.020	0.643	0.074	0.014
**Item 12**	0.100	0.615	0.157	0.008	0.411
**Item 13**	0.241	0.047	0.723	0.263	0.157
**Item 14**	0.051	0.786	0.155	−0.092	0.011
**Item 15**	0.174	0.191	0.618	0.168	−0.249
**Item 16**	0.104	0.304	0.404	0.438	−0.299
**Item 17**	−0.041	0.805	−0.025	0.134	−0.093
**Item 18**	0.183	0.105	0.121	0.722	−0.010
**Item 19**	0.125	0.016	0.337	0.664	0.121
**Item 20**	0.249	0.016	0.060	0.749	0.068
**Item 21**	0.403	−0.072	0.105	0.475	0.282
**Item 22**	0.248	0.531	0.082	0.029	0.369
**Item 23**	0.664	0.018	−0.029	0.325	−0.058
**Item 24**	0.141	0.693	−0.014	−0.023	0.011
**Item 25**	0.480	−0.096	0.319	0.250	0.234

Note: Loadings ≥|0.40| are bold. Component signs are arbitrary and were oriented for interpretability. C2 is dominated by reverse-keyed items (3, 5, 12, 14, 17, 22, 24), indicating a wording-sensitive method component. Total variance explained by the rotated five-component solution was 55.2%.
